# Development of Oral Sustained Release Rifampicin Loaded Chitosan Nanoparticles by Design of Experiment

**DOI:** 10.1155/2013/370938

**Published:** 2013-08-18

**Authors:** Bhavin K. Patel, Rajesh H. Parikh, Pooja S. Aboti

**Affiliations:** Department of Pharmaceutics and Pharmaceutical Technology, Ramanbhai Patel College of Pharmacy, Charotar University of Science and Technology, CHARUSAT Campus, Petlad, Anand, Gujarat 388421, India

## Abstract

*Objective*. The main objective of the present investigation was to develop and optimize oral sustained release Chitosan nanoparticles (CNs) of rifampicin by design of experiment (DOE). *Methodology*. CNs were prepared by modified emulsion ionic gelation technique. Here, inclusion of hydrophobic drug moiety in the hydrophilic matrix of polymer is applied for rifampicin delivery using CN. The 2^3^ full-factorial design was employed by selecting the independent variables such as Chitosan concentration (*X*_1_), concentration of tripolyphosphate (*X*_2_), and homogenization speed (*X*_3_) in order to achieve desired particle size with maximum percent entrapment efficiency and drug loading. The design was validated by checkpoint analysis, and formulation was optimized using the desirability function. *Results*. Particle size, drug entrapment efficiency, and drug loading for the optimized batch were found to be 221.9 nm, 44.17 ± 1.98% W/W, and 42.96 ± 2.91% W/W, respectively. In vitro release data of optimized formulation showed an initial burst followed by slow sustained drug release. Kinetic drug release from CNs was best fitted to Higuchi model. *Conclusion*. Design of Experiment is an important tool for obtaining desired characteristics of rifampicin loaded CNs. In vitro study suggests that oral sustained release CNs might be an effective drug delivery system for tuberculosis.

## 1. Introduction

In spite of the absolute number of incident TB cases falling globally, tuberculosis (TB) continues to be the leading cause of mortality worldwide and has also been considered to be an occupational disease in the health care setup [1]. One of the major problems in the current treatment of tuberculosis is the noncompliance to prescribed regimens, primarily because treatment of TB involves continuous, frequent multiple drug dosing. Adherence to treatment and the outcome of therapy could be improved with the introduction of long-duration drug formulations releasing the antitubercular agents in a slow and sustained manner [2]. Polymer-based drug delivery systems like polymeric nanoparticles have achieved a potential position in the controlled release of therapeutic agents [3]. Polymeric nanoparticles are solid colloidal particles with diameters ranging from 1 to 1000 nm [4]. They consist of macromolecular materials in which the active ingredient is dissolved, entrapped, encapsulated, and adsorbed or chemically attached. 

The fate of nanoparticles in the gastrointestinal tract has extensively been investigated [5–7]. Sustained release cross-linked polymeric nanoparticles enable improvement of drug bioavailability by offering protection to the drugs in gastrointestinal environment and enhancement of solubility because of nanonization. This approach may help in overcoming the first pass effect by getting absorbed from the intestinal tract and entering into the blood streams. Here, the uptake of polymeric nanoparticles may occur by transcytosis via M cells and intracellular uptake and transport via the epithelial cells lining of the intestinal mucosa via Peyer's patches.

The selection of polymer to develop polymeric nanoparticles is dependent on many factors like size of nanoparticles required, inherent properties of the drug, surface characteristics, biodegradability, biocompatibility, toxicity, and drug release desired profile [8]. Chitosan is the most extensively studied polysaccharide to develop polymeric Nanoparticles [9]. As a biodegradable polymer, Chitosan is a popular choice in the application as a drug delivery carrier due to its biocompatibility, chemical versatility, and low cost [10]. In the present study, rifampicin is used as a model antitubercular agent. The main objective of the present study was to formulate and optimize oral sustained release Chitosan nanoparticles of Rifampicin by design of experiment (DOE).

## 2. Materials and Methods

### 2.1. Materials

Chitosan (CS) (degree of deacetylation: 93%) was purchased from Yarrow Chem Products (Mumbai, India). Sodium tripolyphosphate (TPP) was sourced from Sigma-Aldrich (Mumbai, India). Rifampicin was a gift from Cadila Pharmaceuticals Ltd. (Ahmedabad, India) and was of pharmacopeial grade. All other chemicals were of analytical grade.

### 2.2. Methods

#### 2.2.1. Experimental Design

In the present study, a 2^3^ full-factorial experimental design was used to optimize formulation and process parameters for the preparation of Chitosan nanoparticles. In order to optimize, the concentration of Chitosan (*X*_1_), speed of homogenization (*X*_2_), and concentration of tripolyphosphate (TPP) (*X*_3_) were selected as independent variables. Each factor was set at a high level and a low level. The actual values and coded values of different variables are given in Table 1. Eight formulations of drug loaded polymeric nanoparticles (CN_1_ to CN_8_) were prepared according to the design as shown in Table 1. The particle size, percentage of encapsulation efficiency, and percentage of drug loading were taken as response parameters.

#### 2.2.2. Preparation of Rifampicin Loaded Chitosan Nanoparticles

The rifampicin loaded Chitosan nanoparticles were prepared by modified ionic gelation method. In this method, first o/w emulsion was prepared and then ionic gelation was done by polyanionic molecule as previously reported by Ajun et al. [11]. Chitosan solutions (25 mL) of different concentrations (1% w/v, 2% w/v) were prepared by dissolving Chitosan in 1% acetic acid under stirring at room temperature. After dissolving completely, Tween-80 (2% v/v) was added as a surfactant. Subsequently, rifampicin (62.5 mg) was dissolved in dichloromethane (2.5 mL), and then this oil phase was added dropwise to the aqueous phase. This addition was accompanied by stirring at different speeds (19,000 RPM, 26,000 RPM) with the help of high-speed homogenizer (D-8si, ART-MICCRA, Germany). Stirring was continued for 5 minutes after the complete addition of the oil phase to the aqueous phase. Later cross-linking of the particles was induced by the drop wise addition of tripolyphosphate (TPP) solutions (10 mL) of different concentration (0.1% w/v, 0.2% w/v) into o/w emulsion under magnetic stirring at 500 rpm. To ensure complete evaporation of dichloromethane, it was kept overnight at 40°C. Nanoparticles were isolated by centrifugation at 13,500 rpm for 20 minutes at 20°C using cooling centrifuge (Sigma 3K30, Germany), and the supernatant was used for the measurement of free rifampicin by UV spectrophotometer (UV 1800, Shimadzu, Japan).

#### 2.2.3. Particle Size Analysis

The particle size of the formulations was determined by laser scattering technique using Malvern nano S90 (Malvern Instruments, UK) after appropriate dilution with double distilled water. Light scattering was measured at 25°C and with an angle of 90°. The particle size distribution is reported as a polydispersity index (PDI). The range for the PDI is from 0 to 1. The values close to zero indicate the homogenous nature of the dispersion and those greater than 0.5 indicate the heterogeneous nature of the dispersion [12]. 

#### 2.2.4. Morphology

The surface characteristics of samples were studied by scanning electron microscopy (SEM) from 1700x to 5200x magnifications. Double sided carbon tape was affixed on aluminum stubs. The powder sample was dispersed in the double distilled water and dispersion drop was put on the slide. Slide was allowed to dry and was placed on the aluminum stubs. The aluminum stubs were placed in the vacuum chamber of a scanning electron microscope (XL 30 ESEM with EDAX, Philips, The Netherlands). The samples were observed for morphological characterization using a gaseous secondary electron detector (XL 30, Philips, Eindhoven, The Netherlands) with working pressure: 0.8 Torr, acceleration voltage: 30.00 KV.

#### 2.2.5. Percentage of Drug Entrapment Efficiency and Percentage of Drug Loading

The entrapment efficiency and drug loading of selected formulation were calculated by the following equation [13]:

(1) $$\begin{array}{c} \%\;\text{Drug}\;\text{encapsulation}\;\text{efficiency}=\frac{D_{a}-D_{s}}{D_{a}}*100,\\ \%\;\text{Drug}\;\text{loading}=\;\frac{D_{a}-D_{s}}{N_{a}}*100, \end{array}$$

where *D*_*a*_ is the total amount of drug added in system, *D*_*s*_ is the amount of drug in supernatant after the centrifugation, and *N*_*a*_ is the total amount of nanoparticles obtained. The amount of drug in supernatant was calculated from concentration values obtained from the calibration curve on spectrophotometric analysis of the samples at 475 nm (Shimadzu UV 1800, Japan).

#### 2.2.6. Statistical Analysis of Responses by Design Expert

Design Expert 8.0.4. (Stat-Ease, Inc., USA) was used for the analysis of the effect of each variable on the designated response. The statistical significance of the difference in particle size, percentage of drug encapsulation, and percentage of drug loading was tested by one-way analysis of variance (ANOVA) using the following polynomial equation (2):

(2) Y=b0+b1X1+b2X2+b3X3+b1b2X1X2+b1b3X1X3+b2b3X2X3+b1b2b3X1X2X3,

where *Y* is the measured response, *b*_0_  is the arithmetic mean response, *b*_1_ is the main effect of Chitosan concentration (*X*_1_), *b*_2_  is the main effect of speed of homogenization (*X*_2_), and  *b*_3_ is the main effect of TPP concentration (*X*_3_);  *b*_1_ *b*_2_,  *b*_1_ *b*_3_,  *b*_2_ *b*_3_, and  *b*_1_ *b*_2_ *b*_3_  are the interactions of the main factors.

The significant response polynomial equations generated by Design Expert were used to validate the statistical design. Quantitative and qualitative contributions of each variable on each of the responses were analyzed. Response surface plots were generated to visualize the simultaneous effect of each variable on each response parameter.

#### 2.2.7. Checkpoint Analysis

A checkpoint analysis was performed to confirm the utility of the established polynomial equation in the preparation of rifampicin loaded Chitosan nanoparticles. Three checkpoint values of independent variables (*X*_1_, *X*_2_, and *X*_3_) were taken and the values of dependent variables were calculated by substituting the values in the respective polynomial equation (7). Rifampicin loaded Chitosan nanoparticles were prepared experimentally by taking the amounts of the independent variables (*X*_1_, *X*_2_, and *X*_3_). Each batch was prepared three times and mean values were determined. Differences of theoretically computed values of dependent variables and the mean values of experimentally obtained value of dependent variables were compared by using Student *t*'s test method.

#### 2.2.8. Selection of Optimized Formulation on the Basis of Desirability Function

The desirability function was used for optimization of the formulation. During the optimization of formulations, the responses have to be combined in order to produce a product of desired characteristics. Optimized nanoparticles should have low-particle size and high percentage of entrapment efficiency and percentage of drug loading. The individual desirability for each response was calculated using the following method [14, 15].

The percentage of drug encapsulation efficiency and percentage of drug loading values were maximized in the optimization procedure, as optimized nanoparticles batch should have high percentage of drug encapsulation efficiency and percentage of drug loading. The desirability functions of these responses were calculated using the following equation:

(3) $$\begin{array}{c} {\text{ID}}_{1}\;\;\text{or}\;\;{\text{ID}}_{2}=\frac{Y_{i}-Y_{\min}}{Y_{\text{target}}-Y_{\min}},\\ {\text{ID}}_{1}\;\;\text{or}\;\;{\text{ID}}_{2}=1\;\text{for}\;Y_{i}>Y_{\text{target}}, \end{array}$$

where ID_1_  is the individual desirability of percentage of drug encapsulation efficiency and ID_2_  is the individual desirability of percentage of drug loading.

The values of *Y*_target_ and *Y*_min⁡_ for percentage of drug encapsulation efficiency are 49.36 and 20.17, the values of *Y*_target_ and *Y*_min⁡_ for percentage of drug loading are 45.17 and 23.05, and *Y*_*i*_ is the individual experimental result.

The particle size value was minimized in the optimization procedure, as optimized nanoparticles batch should have low particle size. The desirability functions of this response were calculated using the following equation:

(4) $$\begin{array}{c} {\text{ID}}_{3}=\frac{Y_{\max}-Y_{i}}{Y_{\max}-Y_{\text{target}}},\\ {\text{ID}}_{3}=1\;\text{for}\;Y_{i}<Y_{\text{target}}, \end{array}$$

where ID_3_ is the individual desirability of particle size.

The values of *Y*_max⁡_  and *Y*_target_  for particle size were 383.3 and 180.5, and *Y*_*i*_ is the individual experimental result.

The overall desirability values were calculated from the individual desirability values by using the following equation:

(5) $$\begin{array}{c} \text{OD}={\left({\text{ID}}_{1}{\text{ID}}_{2}{\text{ID}}_{3}\cdots {\text{ID}}_{n}\right)}^{1/n}\;, \end{array}$$

where *n* = 3 (number of desirable responses of the experiment).

#### 2.2.9. In Vitro Drug Release

In vitro drug release study of polymeric nanoparticles of the best two batches according to desirability function was performed by the dialysis bag diffusion technique. Polymeric nanoparticles equivalent to 25 mg rifampicin were filled in dialysis bag (MWCO 12–14 kDa, pore size 2.4 nm) and immersed in a receptor compartment containing 150 mL of phosphate buffer solution at three different pH values, 6.8, 5.2, and 7.4, in the presence of ascorbic acid (0.2% w/v). Ascorbic acid was used to prevent the degradation of rifampicin in the dissolution medium due to atmospheric oxygen [16]. The system was stirred at 100 rpm and maintained at a temperature of 37 ± 0.5°C. The pH values were selected to simulate intestinal fluid pH (6.8), physiological pH (7.4), and endosomal pH of macrophages (5.2). At predetermined time intervals, five milliliter of samples was withdrawn and diluted appropriately, and the absorbance was measured by UV/visible spectrophotometer (UV 1800, Shimadzu, Japan) at 475 nm [16]. The results of in vitro drug release were analyzed using model dependent approach. Various kinetic models—zero order, first order, Higuchi, Hixson Crowell and Korsmeyer-Peppas, and Weibull models—were applied to obtain the drug release mechanism from the Chitosan nanoparticles [17–19].

## 3. Result and Discussions

### 3.1. Particle Sizes

Particle sizes of respective batches are shown in Table 1. Particle size was varied in the range of 180.5 (CN_3_) nm to 383.3 (CN_6_). The drug loaded nanoparticles exhibited relatively narrow particle size distribution as indicated by relatively low PDI values in the range of 0.202 to 0.472. Low PDI values also indicate the relative homogenous nature of the dispersion.

### 3.2. Morphology

**Figure 1:**
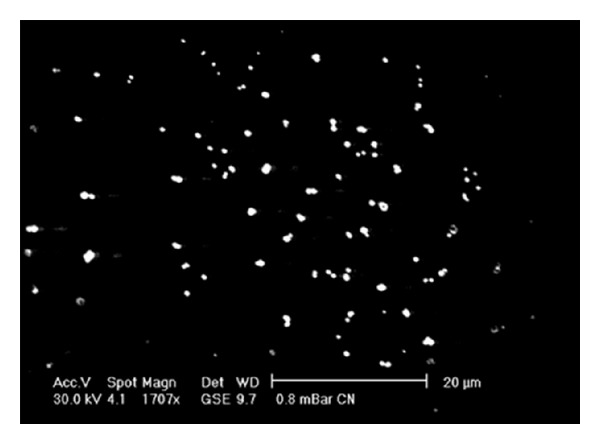
Scanning electron microscope image of Chitosan nanoparticles.

Morphology of chitosan nanoparticles under scanning electron microscope (SEM) is shown in Figure 1. SEM micrograph shows that the Chitosan nanoparticles have regular and uniform spherical shapes. It also shows that there is only little aggregation between the prepared Chitosan nanoparticles. 

### 3.3. Drug Encapsulation Efficiency and Drug Loading

Percentage of drug encapsulation efficiency and percentage of drug loading for respective batches are shown in Table 1. Higher drug encapsulation efficiency and drug loading were observed for the batch CN_8_, and CN_5_ has the lowest drug encapsulation efficiency and drug loading. 

### 3.4. Statistical Analysis of Data

A statistical design was utilized in order to derive the relationship between the response variables and the independent variables. Table 1 shows the independent factors and response values of respective batches. The statistical evaluation of the results was carried out by Design Expert software. The analysis of variance (ANOVA) results (*P* value) of the effect of the variables on particles size, percentage of drug encapsulation efficiency, and percentage of drug loading can be seen in following full-model polynomial equation:

(6) Y1=249.61+31.99X1(P<0.0001)−22.89X2(P<0.0001)+38.39X3(P<0.0001)−8.66X1X2(P<0.0001)+10.76X1X3(P<0.0001)−12.86X2X3(P<0.0001)−8.14X1X2X3(P<0.0001),Y2=29.84+9.92X1(P<0.0001)−2.48X2(P<0.0001)+4.41X3(P=0.0105)−1.77X1X2(P=  0.0551)+1.28X1X3(P=0.1539)+3.61X2X3(P<0.0007)+1.93X1X2X3(P=0.0389),Y3=30.56+8.40X1(P<0.0001)−2.82X2(P=0.0008)+3.89X3(P<0.0084)−1.21X1X2(P=0.2164)+1.67X1X3(P=0.0941)+4.02X2X3(P<0.0006)+1.59X1X2X3(P=0.1108).

The terms of full-model polynomial equation having insignificant *P* value (*P* > 0.05) have negligible contribution to obtained dependent variables and thus are omitted to get reduced model equation. Equations (7) representing the quantitative effect of the formulation and process variables on the particle size, percentage of drug encapsulation efficiency, and percentage of drug loading are described as follows:

(7) Y1=249.61+31.99X1−22.89X2+38.39X3−8.66X1X2+10.76X1X3−12.86X2X3  −8.14X1X2X3;  R2=0.999,Y2=29.84+9.92X1−2.48X2+4.41X3+3.61X2X3+1.93X1X2X3;  R2=0.925,Y3=30.56+8.40X1−2.82X2+3.89X3  +4.02X2X3;  R2=0.892.

Response surface graphs were generated using the above polynomial equations, which represent the simultaneous effect of any two variables on response parameters by taking one variable at a constant level.

Coefficients with one factor in polynomial equations are attributed to the effect of that particular factor, while the coefficients with more than one factor are attributed to the interaction between those factors. A positive sign of the polynomial terms indicates a positive effect, while a negative sign indicates a negative effect of the independent factors.

### 3.5. Effect of Independent Parameters on Dependent Parameters

Polynomial equation (7) represents the effect on particle size, percentage of drug encapsulation efficiency, and percentage of drug loading, respectively. The higher coefficient value of the main effects and interaction terms in the polynomial equation indicates that the effect of independent parameters on particle size is much higher than the effect on percentage of drug encapsulation efficiency and percentage of drug loading. 

**Figure 2:**
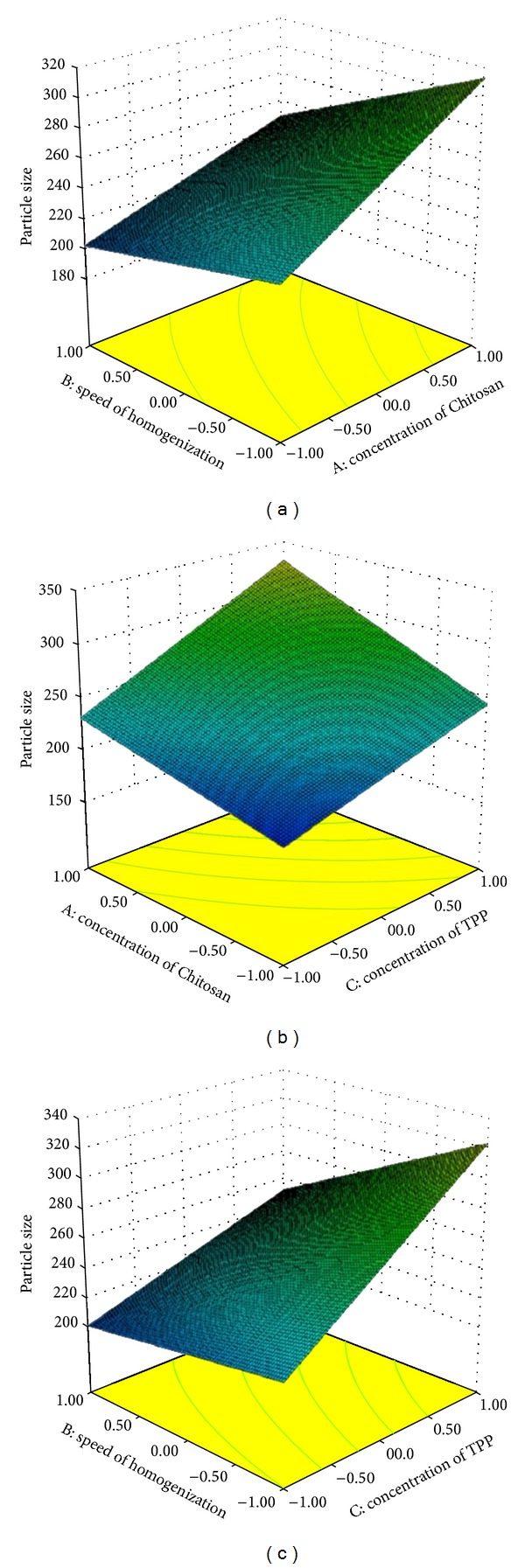
Response surface methodology for the effect of independent parameters on particle size.

**Figure 3:**
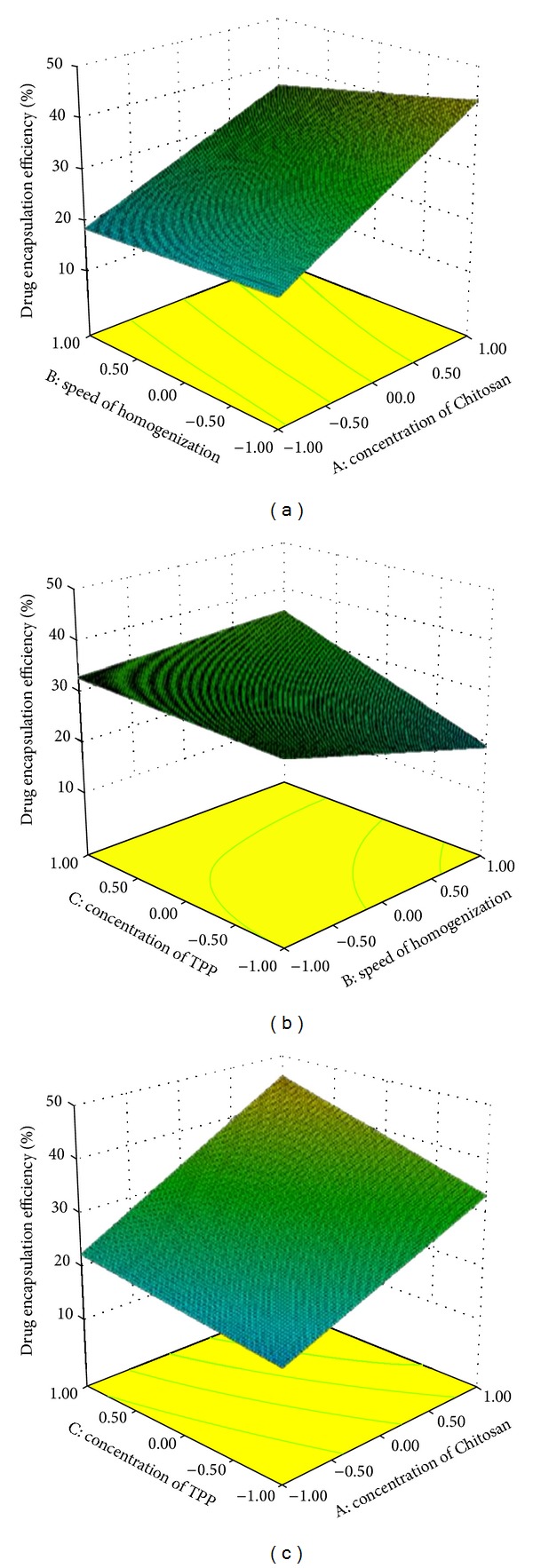
Response surface methodology for the effect of independent parameters on percentage of drug entrapment efficiency.

**Figure 4:**
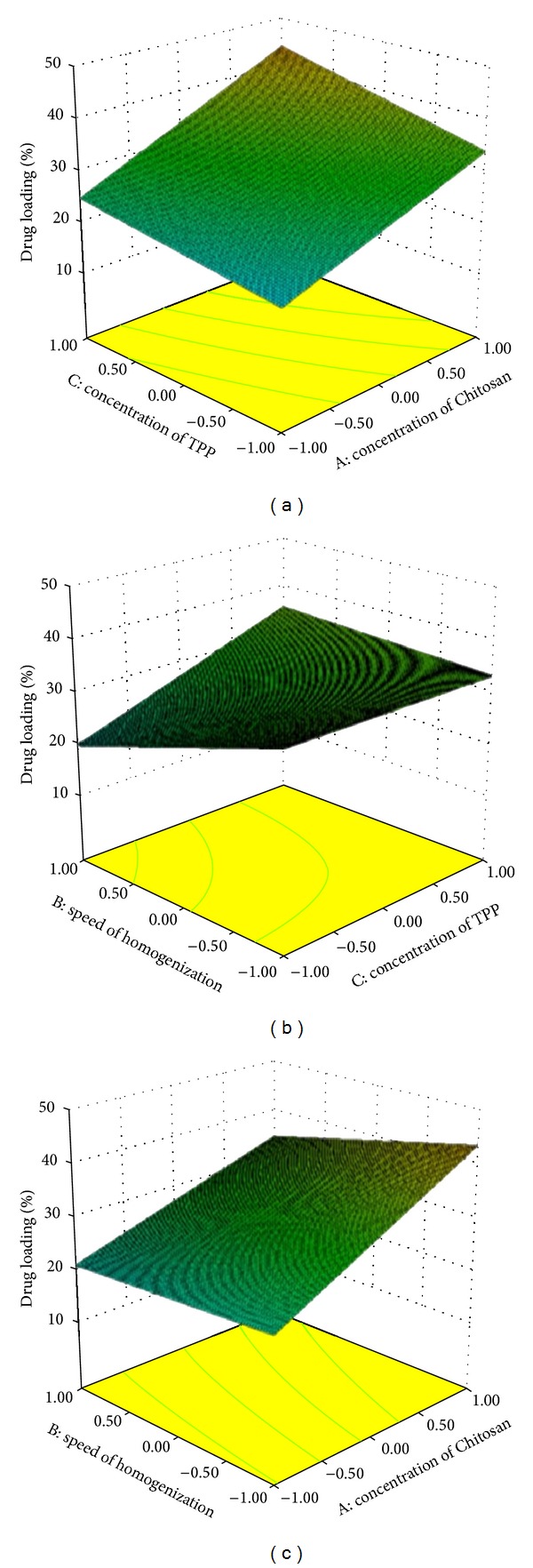
Response surface methodology for the effect of independent parameters on percentage of drug loading.

It can also be concluded that the concentration of Chitosan and concentration of TPP have positive effect; however, the speed of homogenization has a negative effect on all dependent variables. This can also be seen in the response surface methodology indicating the effect of independent parameters on particle size (Figure 2), drug encapsulation efficiency (Figure 3), and drug loading (Figure 4). 

The increase in the particle size with an increase in the concentration of Chitosan is due to the fact that at higher concentration of Chitosan, viscosity is much higher and hence it affects the shear capacity of homogenizer and stirrer as well. The reason for the increases in the particle size with an increase in the concentration of TPP would be due to the stiffness of the cross-linkage between TPP and Chitosan; as the TPP concentration increases, there would be more tripolyphosphoric ions to cross-link with amino groups on Chitosan chains [20]. However, the increase in homogenization speed would decrease particle size, probably due to the fact that at the higher speed, smaller emulsion droplet was formed, resulting in smaller sized particles. 

Increase in the encapsulation efficiency and drug loading with increase of Chitosan concentration would be due to the fact that the higher amount of Chitosan has higher ability of ionic gel formation which prevents the rifampicin movement to the external phase and increases in the drug encapsulation efficiency hence the drug loading. Drug loading and encapsulation efficiency increase with the increase in TPP concentration indicating the better cross-linking density of Chitosan matrix [15]. In addition, at higher speed of homogenization there is a reduction in drug encapsulation efficiency and drug loading. It would be due to diffusion of the drug to the outer phase during emulsification by size reduction using high speed homogenizer [21]. 

### 3.6. Checkpoint Analysis

In order to validate the equation that describes the influence of the factors on the particle size, percentage of drug encapsulation efficiency, percentage of drug loading of nanoparticles, three additional checkpoint experiments (batch CP_1_, batch CP_2_, and batch CP_3_) were taken and Table 2 shows the actual and predicted values of independent parameters. The *t*-test was applied between the actual and predicted values of independent parameters and it was observed that *P* value >0.05. Therefore, it is concluded that the polynomial equations are valid to prepare Chitosan nanoparticles of desired characteristics.

### 3.7. Desirability Function

Desirability function was utilized to identify the best batch out of 8 batches. Table 1 shows the overall desirability value for the respective batches. Batch CN_4_ showed the highest overall desirability of 0.856. Therefore, this batch was considered as the best batch and the values of independent variables of this batch were considered to be optimum values to prepare Chitosan nanoparticles. 

### 3.8. In Vitro Release Study

**Figure 5:**
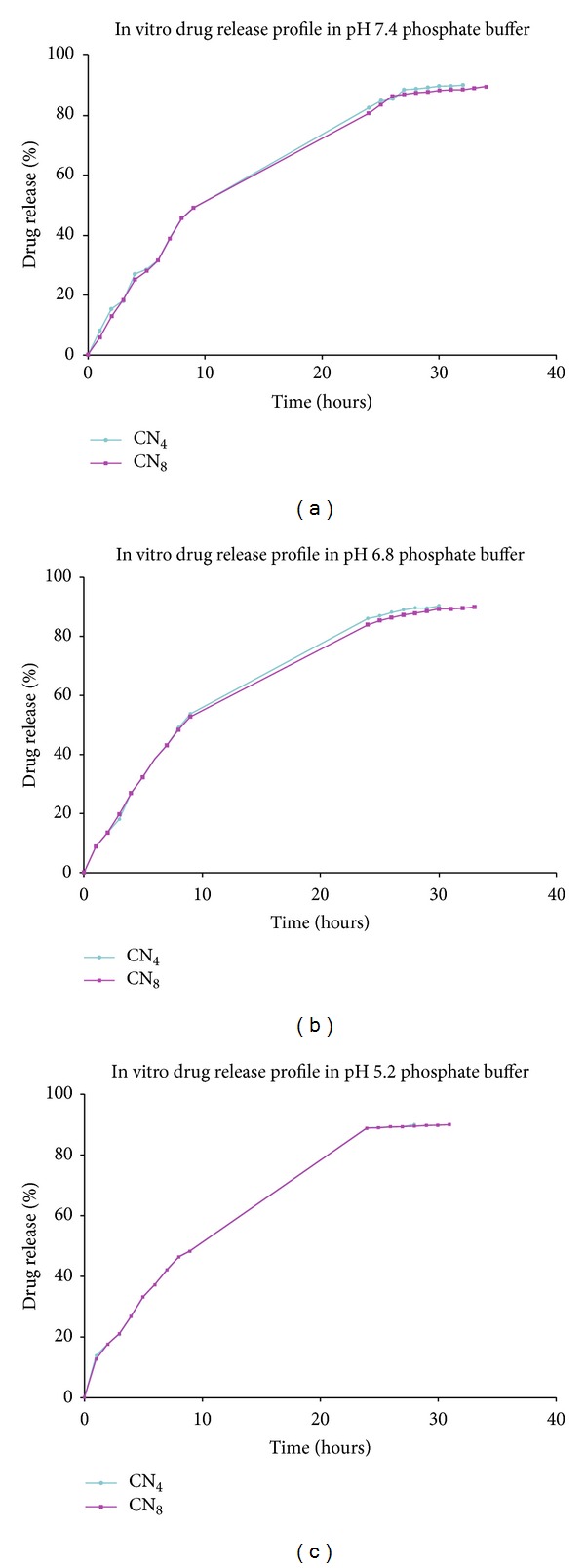
In vitro drug release study of Chitosan nanoparticles.

Release studies were carried out by using three different release medium, phosphate buffers at pH 7.4, pH 6.8, and pH 5.2 in order to simulate the physiological condition, intestinal condition, and the macrophage environment, respectively, shown in Figure 5. At pH 7.4, in both of the batches, about 5% to 8% of the drug is immediately released in 1 hour. Similarly, at pH 6.8 and pH 5.2, in both of the batches, about 8% to 13% of the drug was immediately released in 1 hour. This finding indicates that some of the drug is localized on the surface of the nanoparticles due to the partition of the drug into the surface-active agent layer adsorbed at the surface of the emulsion droplets. After this initial burst, drug release is almost constant, and around 90% of the drug was released from the Chitosan nanoparticles in the range of 28 hours to 34 hours.

It is concluded that rifampicin release of the Chitosan nanoparticles is pH dependent: it is faster at a lower pH than around neutral pH (pH 5.2 > pH 6.8 > pH 7.4). The present work supports the study conducted by Mehta et al. [22]. This is the consequence of the higher solubility of Chitosan at lower pH, where the D-Glucosamine residues are ionized resulting in an extensive polymer swelling and faster drug release. Moreover, rifampicin solubility is pH dependent: it increases as the pH increases. 

When comparing the drug release profiles from CN_8_ and CN_4_ Chitosan nanoparticles, decrease of the release rate is obtained from the cross-linked nanoparticles. This is due to the higher amount of TPP, and hence high degree of cross-linking in the case of CN_8_ compared with that of the  CN_4_. The Higuchi model was best fitted as a release kinetic of Rifampicin from Chitosan nanoparticles.

## 4. Conclusion

Optimization of formulation and process parameters for the development of Chitosan nanoparticles is a prerequisite to obtain the drug loaded Chitosan nanoparticles with desired characteristics. Chitosan nanoparticles were modified by various factors to control particle size, percentage of drug loading, and encapsulation efficiency. The result shows that concentrations of Chitosan, concentration of TPP, and homogenization speed are significantly affecting the particle size, drug loading, and drug encapsulation efficiency. Though rifampicin is a poorly water soluble drug, it can be loaded successfully to a hydrophilic matrix of Chitosan nanoparticles using modified emulsion ionic gelation method. Release of rifampicin from Chitosan nanoparticles was concentration independent and sustains for a longer period of time. Thus, in vivo study can further explore the potentiality of this system for improving patient compliance by reducing the dosing frequencies in tuberculosis.

## Figures and Tables

**Table:** (a)

Batch code	Independent variables			Dependent factors (response value)			Overall desirability (OD)
	*X*_1_^a^	*X*_2_^b^	*X*_3_^c^	Average particle size (nm) (*Y*_1_)	% drug entrapment efficiency (*Y*_2_)	% drug loading (*Y*_3_)	
CN_1_	−1	−1	−1	199.5	20.17 ± 6.53	23.05 ± 8.19	0.3323
CN_2_	+1	−1	−1	243.0	42.89 ± 1.93	43.96 ± 2.33	0.8148
CN_3_	−1	+1	−1	180.5	22.07 ± 1.98	23.56 ± 2.74	0.3860
CN_4_	+1	+1	−1	221.9	44.17 ± 1.98	42.96 ± 2.91	0.8558
CN_5_	−1	−1	+1	264.2	14.40 ± 5.48	15.90 ± 5.82	0.0002
CN_6_	+1	−1	+1	383.3	24.27 ± 2.73	23.78 ± 1.75	0.0000
CN_7_	−1	+1	+1	226.3	23.03 ± 4.07	26.12 ± 4.14	0.4061
CN_8_	+1	+1	+1	278.2	49.36 ± 5.19	45.17 ± 5.15	0.8034

**Table:** (b)

Variables	Levels	
	Low (−1)	High (+1)
*X*_1_^a^	1	2
*X*_2_^b^	19,000	26,000
*X*_3_^c^	0.1	0.2

^a^Concentration of Chitosan (%w/v), ^b^speed of homogenization (rpm), and ^c^concentration of TPP (%w/v).

**Table 2:** Actual and predicted values of dependent variables for checkpoint batch.

Checkpoint batch code	Particle size (nm)		% drug encapsulation efficiency		% drug loading	
	Actual value	Predicted value	Actual value	Predicted value	Actual value	Predicted value
CP_1_	281.1	269.65	35.45	36.90	34.55	36.3
CP_2_	243.3	249.61	31.33	29.84	29.11	30.56
CP_3_	208.4	224.19	23.67	21.59	23.89	26.83
